# Rugged bialkali photocathodes encapsulated with graphene and thin metal film

**DOI:** 10.1038/s41598-023-29374-6

**Published:** 2023-02-10

**Authors:** Lei Guo, Fangze Liu, Kazuki Koyama, Nolan Regis, Anna M. Alexander, Gaoxue Wang, Jeffrey DeFazio, James A. Valdez, Anju Poudel, Masahiro Yamamoto, Nathan A. Moody, Yoshifumi Takashima, Hisato Yamaguchi

**Affiliations:** 1 Nagoya University Synchrotron Radiation Research Center (NUSR), Furo, Chikusa, Nagoya, Aichi 464-8601 Japan; 2 School of Engineering/Graduate School of Engineering, Nagoya University, Furo, Chikusa, Nagoya, Aichi 464-8601 Japan; 3 Advanced Research Institute of Multidisciplinary Sciences, Beijing Institute of Technology, Beijing, 100081 China; 4 Los Alamos National Laboratory (LANL), P.O. Box 1663, Los Alamos, NM 87545 USA; 5 Photonis Defense Inc., 1000 New Holland Ave., Lancaster, PA 17601 USA; 6 Innovation Center for Applied Superconducting Accelerators, High Energy Accelerator Research Organization (KEK), 1-1 Oho, Tsukuba, Ibaraki 305-0801 Japan

**Keywords:** Nanoscience and technology, Graphene

## Abstract

Protection of free-electron sources has been technically challenging due to lack of materials that transmit electrons while preventing corrosive gas molecules. Two-dimensional materials uniquely possess both of required properties. Here, we report three orders of magnitude increase in active pressure and factor of two enhancement in the lifetime of high quantum efficiency (QE) bialkali photocathodes (cesium potassium antimonide (CsK_2_Sb)) by encapsulating them in graphene and thin nickel (Ni) film. The photoelectrons were extracted through the graphene protection layer in a reflection mode, and we achieved QE of ~ 0.17% at ~ 3.4 eV, 1/e lifetime of 188 h with average current of 8.6 nA under continuous illumination, and no decrease of QE at the pressure of as high as ~ 1 × 10^–3^ Pa. In comparison, the QE decreased drastically at 10^–6^ Pa for bare, non-protected CsK_2_Sb photocathodes and their 1/e lifetime under continuous illumination was ~ 48 h. We attributed the improvements to the gas impermeability and photoelectron transparency of graphene.

## Introduction

Bialkali antimonides have been established as high quantum efficiency (QE) semiconductor photocathodes since their discovery in the 1950s. Historically, they have been widely utilized as photosensitive materials in radiation detectors and camera tube technologies^1,2^. In recent years, however, there has been growing interest in their use as high-brightness photocathodes for advanced accelerators and electron microscopes. The next-generation accelerators require high-brightness electron bunches in short pulses that cannot be achieved with thermionic or field emission electron sources. Cesium potassium antimonide (CsK_2_Sb) is one of the best performing photocathodes available with a good balance between QE and the operation pressure^3–15^.The QE can exceed 10% at 532 nm and operation pressure is at 10^–8^ pascals (Pa). Metal photocathodes are the most robust type known, but the QE is generally in 10^–3^–10^–4^% with an exception of cleaned magnesium (Mg) that can reach as high as 0.3% at ~ 250 nm^16^ and cannot be driven by a high-power green laser. Cesiation of surfaces such as gallium arsenide (GaAs) and metals is an alternative, however, extreme ultrahigh vacuum of ~ 10^–9^ Pa is necessary to maintain the QE^17^. The promise of CsK_2_Sb as an electron source for accelerators is supported by the demonstration of record-high beam currents of 60 mA in a DC injector with 30 h 1/e lifetime^5^.

The remaining technical challenges for implementing these photocathodes in accelerators are their low operation pressure of 10^–8^ Pa and short operation lifetime. Increasing the operation pressure and extending the lifetime of bialkali antimonide photocathodes will reduce the number of replacements needed and the time lost for tedious photocathode replacement procedures (including a weeks-long baking of the system). Furthermore, the increased operation pressure will broaden the types of accelerators that bialkali antimonide photocathodes can operate in. There had been previous attempts to protect free-electron sources. Around year 2000, there were a collection of studies to coat bialkali photocathodes with 10–20 nm of more robust materials such as sodium iodide (NaI), cesium iodide (CsI) and cesium bromide (CsBr)^18–21^. While they succeeded in increasing the operation pressure, the thickness of the coating was similar to the thin photocathode thickness itself thus the concept was rather hetero-structuring than coating. With an advancement of two-dimensional (2D) material research over the past decade or so^22,23^, it is timely to apply the coating concept again with 2D materials. The 2D materials uniquely possess critical material properties for the purpose; namely, electron/photon transmissivity and gas impermeability. Graphene for example, transmits electrons due to its atomic thinness while being impermeable to gases due to its packed hexagonal atomic structure. Its optical transparency for monolayer is as high as 97.5% in a visible range^24^. The geometric pore size of graphene is 0.64 Å, which make it impermeable to even helium (He) atoms^25–29^. In addition, graphene is chemically inert and electrically/mechanically stable, which are all advantageous as a protection coating layer.

In this study, we sandwiched CsK_2_Sb photocathodes between graphene and a thin metal film to evaluate the feasibility of using graphene as a protective layer for bialkali antimonides. Specifically, we deposited photocathodes on a free-standing bilayer of graphene and sealed them with a 15–35 nm thick Ni films. Photoelectrons were then extracted through the graphene protection layer in a reflection mode, and the photocathode lifetime under continuous light irradiation was investigated at 10^–8^ Pa. Lifetime of photocathodes without protection was also measured to provide a comparison. We followed the lifetime measurements with active pressure measurements, which measured the QE as a function of pressure to gain insight into whether the graphene protection can increase the operation pressure of CsK_2_Sb photocathodes or not.

## Results and discussion

### Selection of the sealing metal

Overall concept of this study was to create sealed CsK_2_Sb photocathodes to evaluate the feasibility of graphene protection layer in improving their active pressure and the lifetime. Our strategy to achieve this was to deposit thin metal films onto CsK_2_Sb photocathodes via thermal deposition from the opposite side of graphene protection layer. We had already established a protocol to fabricate CsK_2_Sb photocathodes on graphene protection layer^30–34^ thus the remaining task was to select an appropriate sealing metal for our purpose. It is well known that substrate material has severe effects on the QE of deposited ~ 20 nm think CsK_2_Sb photocathodes^8^, therefore it was crucial for us to identify a metal with minimal degradation. We tested copper (Cu), gold (Au) and Ni as metal substrates. Our QE testing method was to deposit CsK_2_Sb photocathodes onto commercially available metal foils rather than depositing thin metal films onto CsK_2_Sb photocathodes. We assumed that this testing method will provide us with necessary information regarding material compatibility between the metals of interest and CsK_2_Sb. The same CsK_2_Sb deposition conditions as that for graphene protection layer was used for consistency (see Method section for details). Cu was chosen for its high thermal conductivity, which could meet the needs to operate photocathodes in cryogenic temperatures such as that for cavity structures. Au was chosen for its chemical inertness, which could prevent photocathodes from interacting with corrosive gases such as oxygen and moisture. Ni was chosen for its potential material compatibility with CsK_2_Sb based on our prior studies^30–34^. We did not test molybdenum (Mo) despite its known material compatibility with CsK_2_Sb because of a concern on its high evaporation temperature. There is a report that the QE of CsK_2_Sb photocathodes start to degrade at temperatures higher than 60 ^o^C^11^ thus we only tested metals with lower evaporation temperatures this time.

**Figure 1:**
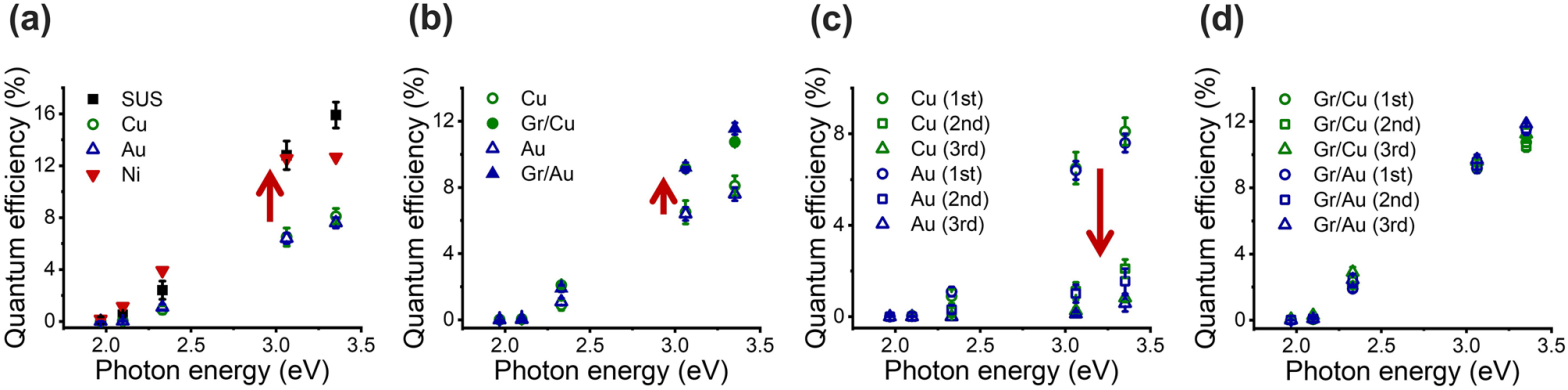
(**a**) QE spectral response of CsK_2_Sb photocathodes on different metal substrates. (**b**) QE spectral response of CsK_2_Sb photocathodes on graphene coated and non-coated metal substrates. (**c**) QE spectral response of CsK_2_Sb photocathodes on metal substrates after repeated depositions. CsK_2_Sb was thermally removed prior to repeated depositions. (**d**) QE spectral response of CsK_2_Sb photocathodes after repeated deposition on graphene-coated metal substrates. Red arrows in (**a**–**c**) indicate directions of the QE changes.

**Figure 2:**
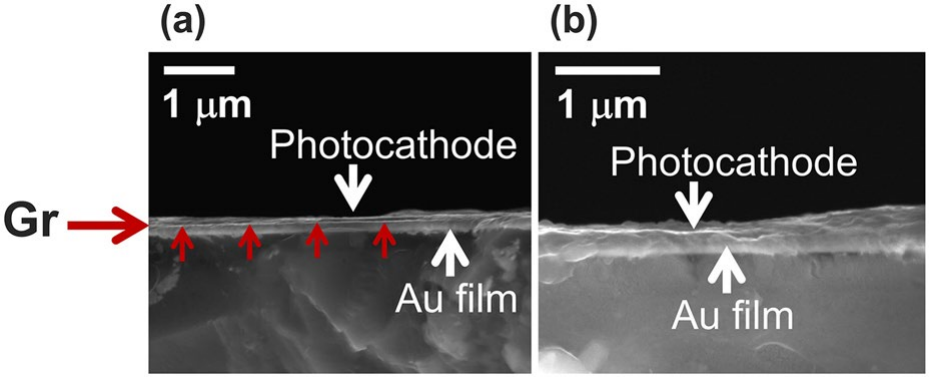
Cross-section SEM images of Cs_3_Sb photocathodes deposited on (**a**) graphene-coated and (**b**) non-coated Au substrates. Images were taken after exposing photocathodes to air thus photocathodes are oxidized. Horizontal and vertical red arrows in (**a**) indicate the location of graphene-coating. White arrows at the top indicate the locations of Cs_3_Sb photocathodes and white arrows at the bottom indicate the locations of Au films on Si substrates. Scale bars are 1 $\upmu$m.

Figure 1a shows the QE spectra of CsK_2_Sb photocathode deposited on Cu, Au, Ni, and stainless steel (SUS) foil substrates at 10^–8^ Pa, where SUS was a reference. It was evident that QE was lower for every metal that we tested in comparison with SUS (12.8% at ~ 3 eV), however, Ni showed a minimal decrease compared to Cu and Au. Specifically, Ni showed the QE of ~ 12.5% (~ 98% of SUS value) whereas the QE for Cu and Au substrates were as low as 6.5% (~ 51% of SUS value) and 6.4% (50% of SUS value), respectively. Prior study of alkali photocathode deposition on Cu substrate reports alloying between Sb and Cu as a possible cause of the QE decrease. They performed *in-situ* synchrotron X-ray diffraction (XRD) and identified peaks associated with Sb-Cu alloy^35^. It is possible this is the cause of our observation on the QE decrease for Cu and Au substrates. The least decrease of QE observed for Ni substrate might indicate that the alloying between CsK_2_Sb is minimal among Cu, Au and Ni. This can be explained by electron affinity of Cu, Au and Ni, which Au has almost twice the value of Cu and the value for Ni is lowest among the three metals. Most direct technique to investigate the alloying between CsK_2_Sb and metal substrates of our interest is to perform XRD. However, this is not trivial as CsK_2_Sb quickly oxidizes upon its exposure to pressure above 10^–6^ Pa during the sample transfers. As an alternative, we coated monolayer graphene on Cu and Au substrates and deposited CsK_2_Sb on top of them to see if there is any improvement in the QE. We hypothesized that if we can reduce the QE decrease via graphene coating, then alloying is playing a role in the QE decrease as graphene is reported to perform well as a diffusion barrier for metals^36,37^. Figure 1b shows the results at 10^–8^ Pa. We observed a clear increase in the QE for CsK_2_Sb photocathodes deposited on graphene-coated Cu and Au substrates in contrast to non-coated counterparts in the dark environment (i.e., at the lower current compared to accelerator facility operation environment). Moreover, graphene-coating prevented a QE decrease of CsK_2_Sb photocathodes even when the substrates were used multiple times. The QE decreased drastically when bare, non-coated Cu and Au substrates were used as substrates for multiple times after thermally cleaning off the photocathode films at 500–600 °C in vacuum for 1 h (Fig. 1c). However, we observed minimal decrease in the QE for CsK_2_Sb photocathode deposited on the graphene-coated substrates (Fig. 1d). This is consistent with our previous observation on CsK_2_Sb photocathode deposited multiple times on graphene-coated and bare, non-coated silicon (Si) and molybdenum (Mo) substrates^31^. In our previous study, synchrotron radiation X-ray photoelectron spectroscopy (SR-XPS) revealed that there was residual photocathode material on Si and Mo substrates even after the thermal cleaning, indicating that there was strong bonding/alloying between the photocathode and the substrates. In sharp contrast, we observed no residue after the cleaning for the graphene-coated substrates, which suggested that the strong bonding/alloying was no longer there. Therefore, the graphene-coating on Cu and Au is most likely acting as a diffusion barrier as expected. Our cross-section scanning electron microscopy (SEM) images on cesium antimonide (Cs_3_Sb) photocathodes deposited on graphene-coated and bare, non-coated Au substrates support the argument (taken after exposure to air). The Cs_3_Sb-Au boundary was sharp for a graphene-coated case (Fig. 2a) whereas it was blur for a non-coated case indicating a mixture/alloying between the two (Fig. 2b).

Our density functional theory (DFT) calculations also supported our hypothesis that graphene coating can prevent alloying between the photocathode elements and metal substrates. We calculated the binding energies between Cs, K and Sb atoms on Cu, graphene-coated Cu and graphene substrates as summarized in Table 1. A negative binding energy indicates the adatom can adsorb on the substrate. We found that Sb atom has the largest binding energies on Cu (111) with −5.54 eV. The binding energies of Cs and K were slightly lower in the range of −2.31 to −2.37 eV. This is in line with the experimental report on alloying formation between Sb and Cu substrate, which decreased the QE of CsK_2_Sb photocathodes^35^. In contrast, the binding energies of Cs, K and Sb decreased to around −1.0 eV on graphene-coated Cu substrate. This is consistent with our experimental observations that QE decrease on Cu and Au substrates compared to that of SUS is reduced by graphene-coating (Fig. 1b). Binding energies for graphene substrate were calculated as references. Based on above experimental and theoretical results, we decided to use Ni as a metal for our sandwich structure to seal CsK_2_Sb photocathodes in between graphene protection layer.

**Table 1:** Binding energies of Cs, K and Sb atoms on Cu, graphene-coated Cu and graphene substrates.

Binding energy (eV)	Cu 111	Gr/Cu 111	Graphene
Cs	− 2.31	− 1.06	− 1.01
K	− 2.37	− 1.12	− 0.87
Sb	− 5.54	− 0.91	− 0.27

### Spectral response photoemission measurements

**Figure 3:**
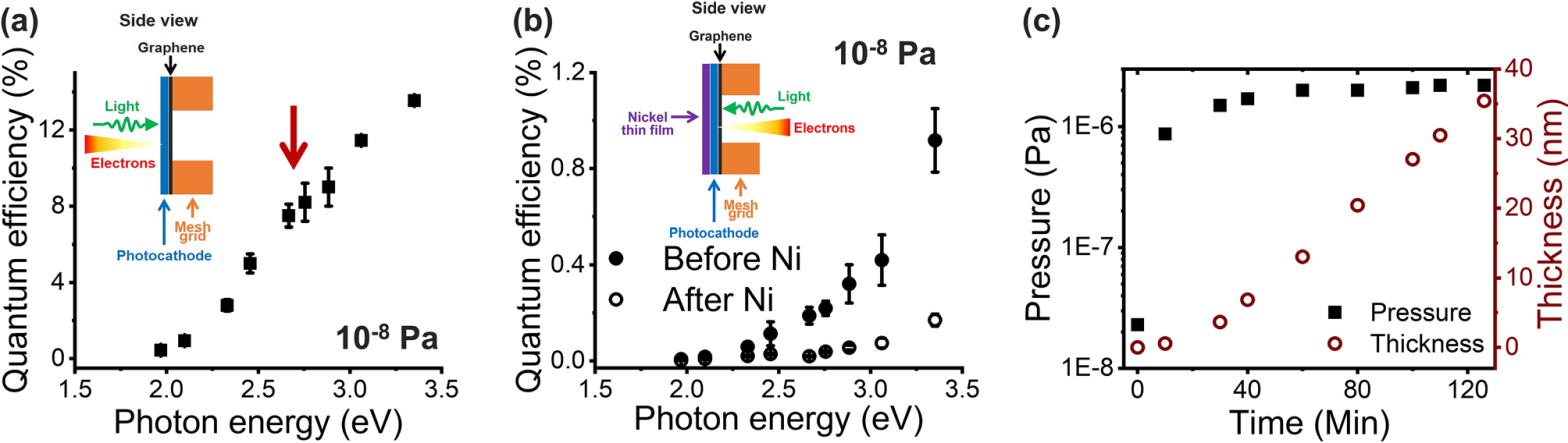
(**a**) QE spectral response of CsK_2_Sb photocathode deposited on TEM-grid-supported bilayer graphene. Inset illustrates an enlarged side view of sample geometry and measurement method for one opening of the TEM-grid. Red arrow indicates the signature hump of CsK_2_Sb photocathodes around ~ 2.5 eV. (**b**) QE spectral response of CsK_2_Sb photocathode deposited on TEM-grid-supported bilayer graphene before and after the Ni seal. Inset illustrates an enlarged side view of sample geometry and measurement method for one opening of the TEM-grid. (**c**) The Ni film thickness-pressure relationship during the Ni deposition. Black squares and red circles are for the pressure and Ni film thickness, respectively.

Figure 3a shows the QE spectral response of CsK_2_Sb photocathodes deposited on transmission electron microscopy (TEM)-grid-supported graphene. The QE was measured in a reflection mode from the CsK_2_Sb side, as illustrated by the inset, and measured at the pressure of 10^–8^ Pa. Successful CsK_2_Sb deposition on graphene is evident from a typical QE response that quickly increases around 2 eV and signature hump around ~ 2.5 eV. Moreover, the peak QE of ~ 13.6% at ~ 3.4 eV is consistent with our previous studies^30–34^.

The QE of CsK_2_Sb photocathodes through bilayer graphene protection layer prior to Ni seal was ~ 0.92% at ~ 3.4 eV, which is consistent with our recent study^30^. Our QE of CsK_2_Sb photocathodes through bilayer graphene protection layer takes TEM-grid transparency into account (see Method section for details). The QE dropped to ~ 0.17% at ~ 3.4 eV after Ni seal, most likely due to the chemisorption and the temperature increase during the Ni deposition (Fig. 3b). 15 nm Ni film was thermally evaporated and the highest pressure during deposition was ~ 2 × 10^–6^ Pa (Fig. 3c). It is likely that partial pressures for corrosive gas species such as moisture and oxygen increased during the deposition, which then chemisorbed onto photocathodes. It is also possible that the temperature at CsK_2_Sb photocathodes increased from the filament for evaporation. Inset of Fig. 3b focuses on one opening of the TEM-grids, but the CsK_2_Sb photocathodes are completely covered thus sealed with Ni thin film including the photocathode edges (see Method section for details). 15 nm thickness was selected based on the knowledge that metals need ~ 15 nm thickness to form uniform and continuous films, which is required for this study to exhibit sufficient seal. Metal films below 15 nm are reported to form islands unless unique treatments are performed on substrate surfaces to control the wettability of metals.

### Lifetime under continuous illumination

**Figure 4:**
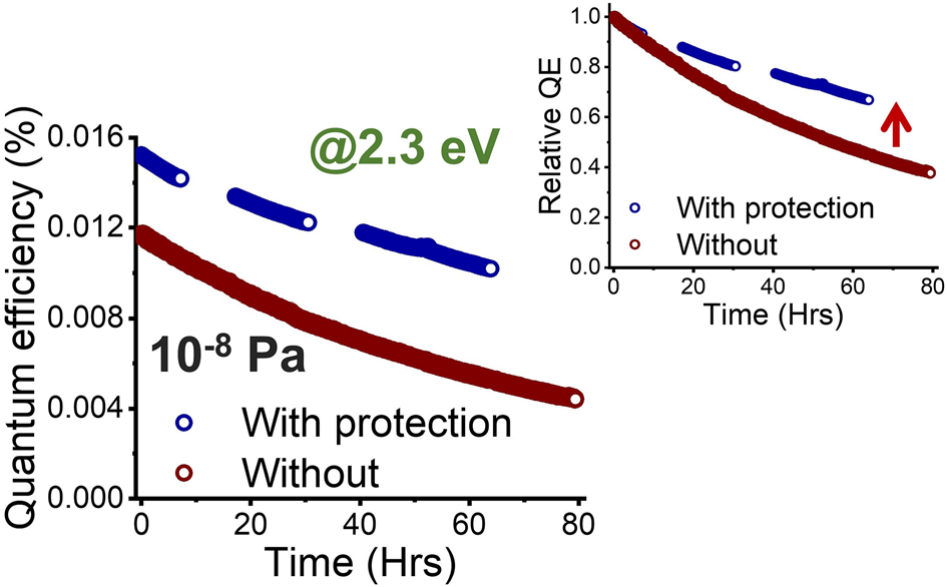
QE evolution over time for bilayer graphene protected (blue) and non-protected (red) CsK_2_Sb photocathodes under a continuous illumination. Inset is normalized comparison with red arrow showing a degree of enhancement.

A set of lifetime tests were performed to investigate the efficacy of the Ni sealed CsK_2_Sb photocathodes. The first test was a lifetime under continuous illumination. The sealed photocathodes were irradiated continuously with a green laser (532 nm) and the QE was measured in a reflection mode through bilayer graphene protection layer under a pressure of 10^–8^ Pa. 1/e lifetime (the time it takes for the QE to decay to ~ 37% of the original value) of 188 h with average current of 8.6 nA was extracted from an exponential curve fitting (Fig. 4). This was factor of two longer than that of the bare, non-protected counterpart at a similar QE (Inset of Fig. 4 shows normalized comparison). The 47.6 h of 1/e lifetime that was observed for bare, non-protected photocathode at as deposited QE (in contrast to Fig. 4, which we waited for the QE to degraded) is consistent with the literature value of 50.7 h measured under very similar conditions^38^. The ~ 2 × improvement demonstrates the potential of our graphene-Ni sandwich strategy to enhance the lifetime of CsK_2_Sb photocathodes in meeting the ~ 2-week turnaround time for feasible operation of accelerators.

The origin of QE decay for bare, non-protected CsK_2_Sb photocathodes is known to be the chemical reactions with oxygen and moisture etc. in the residual gas, which are often combined with ion back bombardments. Ion back bombardments occur when ions generated by the accelerated electrons bombard back to the photocathode surfaces due to the electric field. The fact that there was a decay in QE even for the sealed photocathodes indicates a possible leakage through the Ni thin film due to insufficient thickness. In order to check this, we increased the Ni film thickness to 25 and 35 nm from 15 nm and performed the lifetime tests. However, 1/e lifetime of 188 h under 10^–8^ Pa did not change. These results suggest other cause(s) as the origin of degradation. Another known source of QE degradation is ion back bombardments^5,39,40^ although the effect may be much smaller compared to more susceptible surfaces such as that of cesiated GaAs. The collection voltage of 300 V can ionize H_2_ and H_2_ is known to permeate through graphene slightly more than other gases such as oxygen and moisture. Thus, it is possible that H^2+^ ion back bombardments are the source of QE decrease in our case. We plan to check this in a separate study by inducing controlled gases, including hydrogen, into the deposition chamber and monitoring the QE degradation.

The challenge—aside from addressing the leakage/ion back bombardment issue—is to achieve high QE. We need the QE in the ~ 1% range for green to meet the demands for high-brightness advanced accelerators because of the commercially available laser power (~ 10 W). One way to address this challenge is to improve the quality of our graphene protection layer. The electron transmission efficiency through our current bilayer graphene is ~ 5%^30^ and there is room for improvement toward the theoretical value of ~50% for monolayer. There are reports that have achieved up to ~ 60% electron transmission through monolayer graphene^41,42^, thus we plan to work on optimizing our material synthesis and device fabrication procedures to ensure the highest possible graphene quality in our next set of study. Ensuring the highest QE from CsK_2_Sb photocathodes is another factor that can be improved upon. The highest reported peak QE of CsK_2_Sb photocathodes can reach > 25% in contrast to the ~ 14% we observed in this study. Protected photocathodes with QE in the ~ 1% range and lifetime in the ~ 2-week range are expected after the above-mentioned issues of leakage, ion back bombardments, graphene material quality, and the QE of CsK_2_Sb photocathodes are solved.

### Active pressure

**Figure 5:**
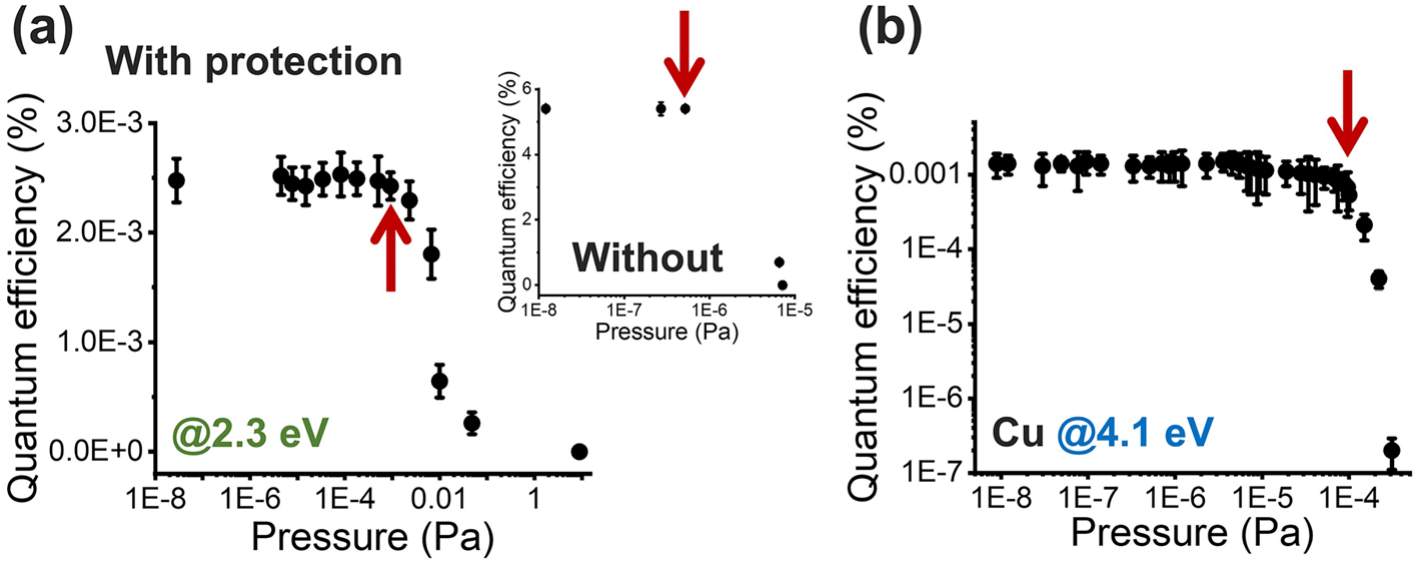
(**a**) Evolution of QE for bilayer graphene protected CsK_2_Sb photocathodes as a function of active pressure. Inset is the QE evolution for non-protected CsK_2_Sb photocathodes at the same photon energy. (**b**) Evolution of QE for polycrystalline Cu photocathodes as a function of active pressure. Red arrow in (**a**,**b**) indicates a pressure, at which the QE starts to decrease drastically.

We followed the lifetime measurements under continuous illumination with active pressure measurements. Specifically, we measured the QE as a function of pressure to find the maximum value at which CsK_2_Sb photocathodes can withstand their QE at average current of 2.4 nA. Similar to the lifetime under continuous illumination case, we irradiated the protected photocathodes with a green laser and measured the QE in a reflection mode through bilayer graphene protection layer. The pressure was gradually increased to atmospheric pressure by inducing air into the vacuum chamber. We observed no decrease of the QE up to ~ 1 × 10^–3^ Pa, which is three orders of magnitude increase in the active pressure of CsK_2_Sb photocathode (Fig. 5a). In comparison, bare, unprotected CsK_2_Sb photocathodes lost their QE drastically when they were exposed to air at ~ 1 × 10^–6^ Pa (Inset of Fig. 5a, measured separately in an identical setup, 10^–8^ Pa was necessary to maintain the QE for long-term). Furthermore, the active pressure of ~ 1 × 10^–3^ Pa for the graphene protected CsK_2_Sb photocathodes was one order of magnitude higher than that of Cu photocathode (~ 1 × 10^–4^ Pa) (Fig. 5b), a type of photocathodes known to be the most robust.

The significance of our achievement is that we succeeded in demonstration of a novel photocathode type using 2D material, graphene protected CsK_2_Sb, that can be excited by a green laser yet active in one order of magnitude higher pressure than metal photocathodes (metal photocathodes cannot be operated with green light). Although the current level in this study is lower than those used in accelerator facility operations, the qualitative implication of our results on the future operation of photocathode components in accelerators can be large. Specifically, we may be able to reduce the replacement time of photocathodes from weeks to a few days. We may no longer need to remove all non-heatable parts for the baking to achieve ultrahigh vacuum pressures of 10^–8^ Pa. The increase of operation pressure will also broaden the types of accelerators bialkali antimonide photocathodes can operate in because of the reduced baking requirement.

**Figure 6:**
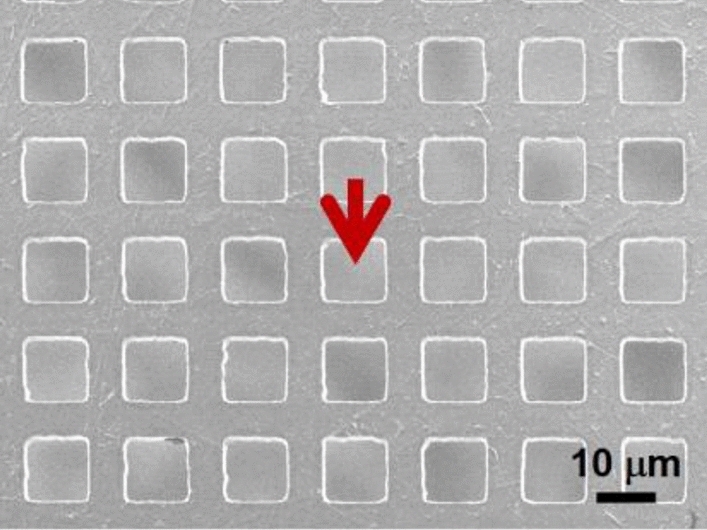
The SEM image of TEM-grid-supported graphene after the photocathode deposition, followed by thermal cleaning/removal at 500–600 °C prior to air exposure. Red arrow indicates a location of graphene spanning over an opening of metal mesh grids.

Our protected photocathodes did not survive the atmospheric pressure. They were physically damaged (i.e., ruptured) when exposed to air. This is most likely due to rapid chemical reactions/oxidation of CsK_2_Sb with oxygen and moisture. We confirmed by SEM that TEM-grid-supported graphene substrates do not rupture upon their exposure to air when CsK_2_Sb was thermally removed at 500–600 °C prior to venting the deposition chamber (Fig. 6).

### Summary discussion

To conclude, we investigated the efficacy of graphene-Ni thin film sandwich structure to seal CsK_2_Sb photocathodes and to improve their active pressure and lifetime under continuous illumination. We achieved an increase of three orders of magnitude in the active pressure and a factor of two enhancements in the lifetime of the protected photocathodes. Specifically, QE of ~ 0.17% at ~ 3.4 eV, 1/e lifetime of 188 h with average current of 8.6 nA under continuous illumination, and no decrease of QE at the pressure of as high as ~ 1 × 10^–3^ Pa were achieved. In comparison, the QE decreased drastically at 10^–6^ Pa for bare, non-protected CsK_2_Sb photocathodes and their 1/e lifetime under continuous illumination was ~ 48 h. Moreover, the active pressure of ~ 1 × 10^–3^ Pa achieved for our protected CsK_2_Sb photocathodes was one order magnitude higher than that of a bare, non-protected Cu photocathode, a type of photocathodes that is known to be the most robust. We attribute the active pressure and lifetime improvements to the gas impermeability and photoelectron transparency of graphene. Our results pave a pathway toward meeting the ~ 2-week turnaround time for feasible operation of accelerators using high-brightness bialkali photocathodes.

## Methods

### Bialkali antimonide photocathode depositions on metal substrates for sealing material selection

Commercially available Cu, Au, Ni and SUS foils were used as purchased (Alfa Aesar and Goodfellow). Those foil substrates were thermally cleaned in ultrahigh vacuum at 500–600 °C for an hour prior to the deposition. CsK_2_Sb photocathode was then deposited onto the cleaned foil substrates. Briefly, the photocathodes were fabricated by sequential thermal evaporation of potassium (K), cesium (Cs), and antimonide (Sb) sources in a vacuum chamber with a base pressure of 1.0 × 10^−8^ Pa. Antimonide was deposited first for ~ 10 nm, followed by ~ 60 nm worth of potassium, and, finally, ~ 120 nm worth of cesium. The exact thicknesses of potassium and cesium were determined by the saturated QE values monitored during the growth using a 532 nm green laser. Detailed experimental setup for CsK_2_Sb photocathode deposition is described in Reference^8^. For cross-section SEM imaging, Cu and Au films with ~ 100 nm thickness was deposited onto Si single crystal substrate with 100 nm oxide layer via electron-gun (e-gun) evaporation.

### Sandwiching bialkali antimonide photocathodes between graphene and nickel thin films

Free-standing graphene was first prepared on commercially available TEM metal mesh grids with a 3 mm diameter. Graphene consisting of mainly monolayer regions with some multiple layer regions was synthesized via chemical vapor deposition (CVD)^43^ on Cu substrates were purchased from Graphenea and transferred onto TEM mesh grids using an established polymer-supported method that involves distilled water exposure^44^. Bilayer graphene, instead of the monolayer, was used to minimize mechanical voids generated during the transfer process. Polymer support was removed using acetone. The graphene on TEM grids were thermally cleaned in ultrahigh vacuum at 500–600 °C for an hour to remove residue from polymer support during transfers. CsK_2_Sb photocathode was then deposited onto the graphene using the same condition as that for sealing metal selection. CsK_2_Sb photocathodes on TEM-grid-supported graphene were lastly sealed by 15–35 nm Ni thin films deposited via thermal evaporation. The deposition rate of Ni thin films was ~ 0.06 Å/s. The evaporation area of Ni thin film was 6 mm diameter circle thus large enough to cover the entire 3 mm diameter TEM-grid with CsK_2_Sb photocathode on graphene and seal it at the edge.

### Photoemission measurements

Reflection mode QE values were measured in vacuum using lasers in the visible wavelengths. The laser spot size was 0.5 mm^2^, and the photocathodes were biased at −100 to −200 V to ensure the effective collection of photoelectrons. The laser power was adjusted to 0.6 mW, and the typical photo-current level was several µA for spectral response (e.g., photo-current of ~ 6.7 µA was achieved for QE of 2.8% at 532 nm) and several nA for lifetime measurements. The QE spectral response for CsK_2_Sb photocathodes on TEM-grid-supported graphene was initially measured from the photocathode and the graphene sides separately. The QE from the graphene side was measured through bilayer graphene protection layer and it was measured before and after Ni thin film seal. In all QE that is measured from graphene side, the TEM-grid transparency of 40% is considered (i.e., raw QE is multiplied by 2.5). The lifetimes of Ni sealed photocathodes were measured from the graphene side, under continuous illumination. A 532 nm green laser was used for all lifetime and operation pressure measurements. The lifetime measurements were performed at 10^−8^ Pa. The pressure was intentionally increased to atmospheric pressure by inducing air for the active pressure measurements.

### DFT calculations

Calculations were performed with the use of density functional theory (DFT) and the projector augmented-wave (PAW) method^45^ as implemented in the Vienna ab initio Simulation Package (VASP)^46^. The generalized gradient approximation (GGA) of the Perdew–Burke–Ernzerhof (PBE)^47^ functional was used to represent the exchange–correlation interaction. The metal substrates were represented with slab models with a vacuum gap in the direction normal to the surface to eliminate the interactions between the replicas due to the periodic boundary conditions. The binding energies of adatoms are defined as ${E}_{\mathrm{b}}={E}_{\mathrm{substrate}+\mathrm{adatom}}-({E}_{\mathrm{substrate}}+ {E}_{\mathrm{adatom}})$, where ${E}_{\mathrm{substrate}+\mathrm{adatom}}$, ${E}_{\mathrm{substrate}}$, and ${E}_{\mathrm{adatom}}$ are the total energies of the complex system, the substrate, and the adatom, respectively. A negative binding energy indicates the adatom can adsorb on the substrate.

## Data Availability

The data that support the findings of this study are available from the corresponding author upon reasonable request.

## References

[1] Sommer AH (1980). Photoemissive Materials.

[2] Carrot WH (1965). Chemical and structural characteristics of the potassium-cesium-antimony photocathode. J. Phys. Chem. Solids.

[3] Parzyck CT (2022). Single-crystal alkali antimonide photocathodes: High efficiency in the ultrathin limit. Phys. Rev. Lett..

[4] Bazarov I (2011). Thermal emittance measurements of a cesium potassium antimonide photocathode. Appl. Phys. Lett..

[5] Dunham B (2013). Record high-average current from a high-brightness photoinjector. Appl. Phys. Lett..

[6] Vecchione T (2011). A low emittance and high efficiency visible light photocathode for high brightness accelerator-based X-ray light sources. Appl. Phys. Lett..

[7] Schubert S (2013). Bi-alkali antimonide photocathodes for high brightness accelerators. APL Mater..

[8] Guo, L., Kuriki, M., Yokota, A., Urano, M. & Negishi, K. Substrate dependence of CsK2Sb photo-cathode performance. *Progr. Theor. Exp. Phys.* **2017**, 033G001–033G001, doi:10.1093/ptep/ptx030 (2017).

[9] Cultrera L (2015). Cold electron beams from cryocooled, alkali antimonide photocathodes. Phys. Rev. Spec. Top. Accel Beams.

[10] Cultrera L, Lee H, Bazarov I (2016). Alkali antimonides photocathodes growth using pure metals evaporation from effusion cells. J. Vac. Sci. Technol. B Nanotechnol. Microelectron. Mater. Process. Measur. Phenomena.

[11] Ding Z (2017). Temperature-dependent quantum efficiency degradation of K-Cs-Sb bialkali antimonide photocathodes grown by a triple-element codeposition method. Phys. Rev. Acceler. Beams.

[12] Feng J (2017). Near atomically smooth alkali antimonide photocathode thin films. J. Appl. Phys..

[13] Ruiz-Osés M (2014). Direct observation of bi-alkali antimonide photocathodes growth via in operando x-ray diffraction studies. APL Mater..

[14] Schubert S (2016). Bi-alkali antimonide photocathode growth: An X-ray diffraction study. J. Appl. Phys..

[15] Guo L, Katoh M (2019). pn-type substrate dependence of CsK2Sb photocathode performance. Phys. Rev. Acceler. Beams.

[16] Xiang, R., Michel, A. A. P., Murcek, P., Teichert, J., Lu, P., Vennekate, H., & Patra, P. in *International Particle Accelerator Conference (IPAC) 2017.* MOPIK003.

[17] Guo L, Kuriki M, Iijima H, Uchida K (2017). NEA surface activation of GaAs photocathode with different gases. Surf. Sci..

[18] Breskin A, Buzulutskov A, Chechik R, Prager M, Shefer E (1996). Evidence for thin-film protection of visible photocathodes. Appl. Phys. Lett..

[19] Shefer E (2002). Photoelectron transport in CsI and CsBr coating films of alkali antimonide and CsI photocathodes. J. Appl. Phys..

[20] Buzulutskov A, Shefer E, Breskin A, Chechik R, Prager M (1997). The protection of K-Cs-Sb photocathodes with CsBr films. Nucl. Instrum. Methods Phys. Res. Sect. A.

[21] Shefer E (1999). Coated photocathodes for visible photon imaging with gaseous photomultipliers. Nucl. Instrum. Methods Phys. Res. Sect. A.

[22] Geim AK, Novoselov KS (2007). The rise of graphene. Nat. Mater..

[23] Geim AK (2009). Graphene: Status and prospects. Science.

[24] Nair RR (2008). Fine structure constant defines visual transparency of graphene. Science.

[25] Leenaerts O, Partoens B, Peeters FM (2008). Graphene: A perfect nanoballoon. Appl. Phys. Lett..

[26] Sun PZ (2020). Limits on gas impermeability of graphene. Nature.

[27] Liu Z, Li J, Yan F (2013). Package-free flexible organic solar cells with graphene top electrodes. Adv. Mater..

[28] Su Y (2014). Impermeable barrier films and protective coatings based on reduced graphene oxide. Nat. Commun..

[29] Huang S (2018). Single-layer graphene membranes by crack-free transfer for gas mixture separation. Nat. Commun..

[30] Liu F (2022). Photoemission from Bialkali Photocathodes through an Atomically Thin Protection Layer. ACS Appl. Mater. Interfaces..

[31] Guo L (2020). Graphene as reusable substrate for bialkali photocathodes. Appl. Phys. Lett..

[32] Yamaguchi H (2019). Quantum efficiency enhancement of bialkali photocathodes by an atomically thin layer on substrates. Physica Status Solidi (a).

[33] Yamaguchi H (2018). Free-standing bialkali photocathodes using atomically thin substrates. Adv. Mater. Interfaces.

[34] Yamaguchi H (2017). Active bialkali photocathodes on free-standing graphene substrates. NPJ 2D Mater. Appl..

[35] Smedley, J., Rao, T., Ben-Zvi, I., Ruiz-Osés, M., Liang, X., Muller, E. M., Lee, S., Attenkofer, K., Padmore, H., & Vecchione, T. in *International Particle Accelerator Conference (IPAC) 2011.* THPC136.

[36] Hong J (2014). Graphene as an atomically thin barrier to Cu diffusion into Si. Nanoscale.

[37] Morrow WK, Pearton SJ, Ren F (2016). Review of graphene as a solid state diffusion barrier. Small.

[38] Schmeißer, M. A. H. *et al.* Towards the operation of Cs-K-Sb photocathodes in superconducting rf photoinjectors. *Phys. Rev. Acceler. Beams* **21**, 113401. 10.1103/PhysRevAccelBeams.21.113401 (2018).

[39] Cultrera, L. *et al.* Photocathode behavior during high current running in the Cornell energy recovery linac photoinjector. *Phys. Rev. Spec. Top. – Acceler. Beams* **14**, 120101. 10.1103/PhysRevSTAB.14.120101 (2011).

[40] Cultrera L (2013). Growth and characterization of rugged sodium potassium antimonide photocathodes for high brilliance photoinjector. Appl. Phys. Lett..

[41] Hassink, G. *et al.* Transparency of graphene for low-energy electrons measured in a vacuum-triode setup. *APL Mater.* **3**, 076106. 10.1063/1.4927406 (2015).

[42] Murakami K (2020). Mechanism of highly efficient electron emission from a graphene/oxide/semiconductor structure. ACS Appl. Electron. Mater..

[43] Li X (2009). Large-area synthesis of high-quality and uniform graphene films on copper foils. Science.

[44] Reina A (2008). Transferring and identification of single- and few-layer graphene on arbitrary substrates. J. Phys. Chem. C.

[45] Kresse G, Joubert D (1999). From ultrasoft pseudopotentials to the projector augmented-wave method. Phys. Rev. B.

[46] Kresse G, Furthmüller J (1996). Efficiency of ab-initio total energy calculations for metals and semiconductors using a plane-wave basis set. Comput. Mater. Sci..

[47] Perdew JP, Burke K, Ernzerhof M (1996). Generalized gradient approximation made simple. Phys. Rev. Lett..

