# Author Correction: Rugged bialkali photocathodes encapsulated with graphene and thin metal film

**DOI:** 10.1038/s41598-023-30799-2

**Published:** 2023-03-02

**Authors:** Lei Guo, Fangze Liu, Kazuki Koyama, Nolan Regis, Anna M. Alexander, Gaoxue Wang, Jeffrey DeFazio, James A. Valdez, Anju Poudel, Masahiro Yamamoto, Nathan A. Moody, Yoshifumi Takashima, Hisato Yamaguchi

**Affiliations:** 1 Nagoya University Synchrotron Radiation Research Center (NUSR), Furo, Chikusa, Nagoya, Aichi 464-8601 Japan; 2 School of Engineering/Graduate School of Engineering, Nagoya University, Furo, Chikusa, Nagoya, Aichi 464-8601 Japan; 3 Advanced Research Institute of Multidisciplinary Sciences, Beijing Institute of Technology, Beijing, 100081 China; 4 Los Alamos National Laboratory (LANL), P.O. Box 1663, Los Alamos, NM 87545 USA; 5 Photonis Defense Inc., 1000 New Holland Ave., Lancaster, PA 17601 USA; 6 Innovation Center for Applied Superconducting Accelerators, High Energy Accelerator Research Organization (KEK), 1‑1 Oho, Tsukuba, Ibaraki 305‑0801 Japan

Correction to: *Scientific Reports* 10.1038/s41598-023-29374-6, published online 10 February 2023

The original version of this Article contained errors.

The Article contained an error in Affiliation 6, which was incorrectly given as ‘Accelerator Division 6, High Energy Accelerator Research Organization (KEK), 1‑1 Oho, Tsukuba, Ibaraki 305‑0801, Japan’. The correct affiliation is listed below:

Innovation Center for Applied Superconducting Accelerators, High Energy Accelerator Research Organization (KEK), 1‑1 Oho, Tsukuba, Ibaraki 305‑0801, Japan

Additionally, the Article contained an error in Results and discussion section, under ‘Selection of the sealing metal’ subheading,

“In our previous study, synchrotron X-ray photoelectron spectroscopy (XPS) revealed that there was residual photocathode material on Si and Mo substrates even after the thermal cleaning, indicating that there was strong bonding/alloying between the photocathode and the substrates.”

now reads:

“In our previous study, synchrotron radiation X-ray photoelectron spectroscopy (SR-XPS) revealed that there was residual photocathode material on Si and Mo substrates even after the thermal cleaning, indicating that there was strong bonding/alloying between the photocathode and the substrates.”

The subheading ‘Photoemission measurement’,

now reads:

“Spectral response photoemission measurements.”

Under ‘Lifetime under continuous illumination’ subheading,

“These results suggest other cause(s) as the origin of leakage.”

now reads:

“These results suggest other cause(s) as the origin of degradation.”

“The electron transmission efficiency through our current bilayer graphene is ~ 5%^30^ and there is room for improvement toward the theoretical value of ~ 50%.”

now reads:

“The electron transmission efficiency through our current bilayer graphene is ~ 5%^30^ and there is room for improvement toward the theoretical value of ~ 50% for monolayer.”

Finally, in the Methods, under the subheading ‘Sandwiching bialkali antimonide photocathodes between graphene and nickel thin films’,

“Graphene consisting of mainly monolayer regions with some multiple layer regions was synthesized via chemical vapor deposition (CVD)^43^ on Cu substrates and transferred onto TEM mesh grids using an established polymer-supported method that involves distilled water exposure^44^. Bilayer graphene, instead of the monolayer, was used to minimize mechanical voids generated during the transfer process.”

now reads:

“Graphene consisting of mainly monolayer regions with some multiple layer regions was synthesized via chemical vapor deposition (CVD)^43^ on Cu substrates were purchased from Graphenea and transferred onto TEM mesh grids using an established polymer-supported method that involves distilled water exposure^44^. Bilayer graphene, instead of the monolayer, was used to minimize mechanical voids generated during the transfer process. Polymer support was removed using acetone.”

The original Article has been corrected.

